# Effect of prolonged critical care admissions on upper and lower limb muscle architecture

**DOI:** 10.1186/cc14553

**Published:** 2015-03-16

**Authors:** P Turton, R Hay, JK Taylor, J Little, J McPhee, I Welters

**Affiliations:** 1 Royal Liverpool and Broadgreen University Hospitals, Liverpool, UK; 2 Warrington and Halton Hospitals NHS Trust, Warrington, UK; 3 Manchester Metropolitan University, Manchester, UK; 4 University of Liverpool, UK

## Introduction

Muscle wasting is a common consequence of long-term stay in the critical care environment, which may slow down the rehabilitation of survivors. Previous ultrasound studies have demonstrated a loss of cross-sectional area of lower limb muscles during a 10-day intensive care stay. In this study, we have looked at how markers of muscle architecture (muscle thickness, pennation angle and fascicle length) change in the lower limb, as well as looking at changes in muscle thickness in the upper limb.

## Methods

Following ethical approval, patients who were intubated and ventilated in one of two critical care departments were assented to take part in the study by their next of kin. B-mode ultrasound scans of the right biceps, vastus lateralis and the medial head of gastrocnemius were performed on days 1, 5 and 10. Scans were not performed in patients once they were free of sedation. Muscle thickness (MT) was measured in all three muscles, with pennation angle (PA) being measured in the lower limb muscles. Fascicle length (FL) was derived from PA and MT.

## Results

Twenty patients were recruited, of which 15 were scanned on day 5, and eight were scanned on day 10. In the biceps, there were no alterations in MT over 5 or 10 days. MT of the vastus lateralis significantly decreased on day 5 (1.77 ± 0.06 mm muscle loss, *P *= 0.03) and day 10 (5.58 ± 0.09 mm muscle loss, *P *= 0.01). There was also a significant loss in PA over 5 days (1.48 ± 0.63°, *P *= 0.01) and 10 days (2.96 ± 0.72°, *P *= 0.01). However, FLwas unchanged over time. There was a significant relationship between size of PA and percentage loss of PA and FLin over 5 days. Loss of MT and PA (MT: 3.21 ± 2.08 mm lost, PA: 2.19 ± 1.64°) was observed in the medial gastrocnemius over 10 days, but did not approach significance. Large fascicles on day 1 were associated with greater percentage loss of FLon day 5 (*P *= 0.012).

## Conclusion

In the lower limb, we have shown that MT and PA alterations occur in the first 10 days. Patients with larger PA and FLappear to lose a greater percentage of angle and fascicle length in the first 5 days. In contrast, we have demonstrated a sparing effect on the muscles of the upper limb compared with the lower limb. These findings may have implications for rehabilitation and interventions to preserve muscle mass.

